# Analyzing nonlinear and asymmetric effects of green finance and renewable energy on energy efficiency amidst technological innovation in E7 countries

**DOI:** 10.1016/j.heliyon.2024.e35895

**Published:** 2024-08-13

**Authors:** Mingda Shi, Jing Yu

**Affiliations:** a Social Hub, Fintech Thrust, Hong Kong University of Science and Technology Guangzhou, Guangzhou, Guangdong Province, 511458, China; b Harbin University, China

**Keywords:** Energy efficiency, Green finance, E7 countries, Renewable energy

## Abstract

The study aims to explore the relationship between green funding, green energy, and energy efficiency in E7 countries, guided by the SDG-7 guidelines recommended by the United Nations General Assembly. Methodologically, the study employs the Nonlinear Autoregressive Distributed Lag (NARDL) and Two-Stage Least Squares (2SLS) techniques on data collected between 1988 and 2022. The rationale for this approach lies in its ability to capture both short-term and long-term dynamics in the relationship between green funding, green energy, and energy efficiency. Analysis of the data reveals varying stages of green funding growth among E7 countries, with China (1.52), Brazil (1.44), India (1.35), Indonesia (1.94), Mexico (1.73), and Russia (1.93) exhibiting different levels of progress. Russia and Turkey are identified as having the highest Gini coefficients in 2019, indicating disparities in green funding distribution within these countries. The empirical findings underscore the critical role of investment in the energy sector by both corporations and the public sector to enhance access to electricity, bolster energy security, and foster environmentally sustainable economic development. However, the study identifies insufficient investment as a fundamental obstacle hindering progress in green energy efficiency in E7 nations. Despite the potential for implementing energy efficiency measures and renewable energy sources in E7 countries, the future remains uncertain due to existing obstacles in green financing and regulatory frameworks. Consequently, the paper emphasizes the imperative of addressing these obstacles to unlock the full funding potential for energy efficiency initiatives in E7 nations.

## 1 Introduction

Over the past decade, E7 nations have made substantial strides toward establishing a reliable and environmentally friendly electricity infrastructure. Greenhouse gas emissions have been steadily decreasing due to measures including the gradual restart of nuclear power plants, an increase in renewable energy sources, and improvements in energy efficiency. However, fossil fuels have played a crucial role in bridging the gap caused by the temporary shutdown of nuclear power facilities in E7 nations. Despite these efforts, the E7 nations remain highly dependent on foreign supplies of natural fuels.

Eighty-eight percent of the world's energy still comes from fossil fuels, and the carbon intensity of that energy has been rising rapidly since 2011. To effectively reduce carbon emissions by 2050, accelerating efforts in low-carbon technology, addressing administrative and regulatory barriers, and enhancing competition in the energy market are all essential for E7 nations [1].

Energy consumption in E7 countries is predominantly fueled by sources that pose security risks due to their greenhouse gas emissions, highlighting the urgent need to enhance energy efficiency [2]. However, grappling with electricity-related challenges is not a new phenomenon for these nations. As a result, they have faced difficulties in meeting energy demands and improving energy efficiency [1].

The role of the finance industry in accelerating the transition to low-carbon energy sources has become increasingly significant [3]. With the establishment of the United Nations' Sustainable Development Goals and the climate targets outlined in theParis Climate Agreement, global funding commitments have surged to an estimated US$100 billion. It is projected that between US$1.6 and US$3.8 trillion will need to be invested annually in energy systems between 2020 and 2050 to maintain effective systems and mitigate adverse climate impacts [4].

These figures underscore the urgent need to transition to clean, low-carbon energy sources. Notably, low-carbon investments and initiatives to support the UN's sustainable development objectives are particularly pronounced in E7 nations. Recently, the E7 council authorized $40 billion to decarbonize energy systems and promote green economic development, highlighting the significance of green funding in facilitating low-carbon energy transitions and improving energy efficiency.

The establishment of the Green Finance Network aims to facilitate communication between public and private sector actors involved in green finance within E7 nations. Green bonds are identified as crucial instruments for enhancing the energy economy, as explained within the framework of ASEAN nations [5].

However, challenges persist, particularly in accessing green funding in China, hindering efforts to optimize energy systems. Nonetheless, there is growing traction for green funding initiatives in the developed economies of the E7, fostering low-carbonization efforts and enhancing energy efficiency.

Furthermore, recent advancements in financial technology (FinTech), especially within E7 nations, are anticipated to significantly contribute to financial inclusion and well-being. These developments underscore the interconnectedness of financial systems and sustainable energy transitions within E7 nations, highlighting the multifaceted approach required to address energy challenges while promoting economic development and environmental sustainability. The delivery and utilization of automated operations and processes to enhance financial services have undergone a revolution due to financial technology (Fintech). Literature reviews underscore the significance of Fintech in poverty alleviation, financial stability promotion, and urban growth facilitation. Moreover, Fintech has facilitated the harnessing of green energy for economic and environmental benefits [6].

[7] advocate for green energy financing through a combination of multilateral financial institutions (MFIs) and internet-based community groups, a strategy supported by Ref. [8] who highlight the rapid advancement potential of new technologies in boosting the energy economy. Additionally [9], argue that Fintech can potentially organize green finance in the future by easing access to new forms of capital and investment.

The transition to a green economy can be further facilitated by Fintech, which plays a pivotal role in providing green funding through the integration of big data and artificial intelligence. Furthermore [10], emphasize that Fintech ensures environmentally friendly financial transactions, while Blockchain technology, an example of Fintech, can overcome market hurdles and enhance energy-saving initiatives.

[11] advocate for the issuance of green bonds to increase capital flow and ensure energy savings as part of green finance. These assertions collectively indicate the potential roles of green finance and Fintech in addressing the escalating demand for energy financing. Additionally [12], provides supporting research, emphasizing the crucial role of Fintech in facilitating green funding.

However, E7 nations have been slower in adopting Fintech compared to countries like the United Kingdom, China, Singapore, and the United States. Nonetheless, studies suggest that E7 nations are considering integrating Fintech into their financial systems to bolster them. Therefore, the combination of green finance and Fintech holds promise in mitigating the economic and environmental impacts of energy-saving challenges in E7 nations.

Economic growth is an integral aspect of economic expansion, and financial integration is crucial [13]. Inclusive financial systems can positively influence environmental practices, fostering the possibility of investing in green technology by providing easy access to financial schemes.

This research addresses several gaps in the existing literature. Firstly, it contributes to the literature on climate funding distribution by examining limitations on the outlay side, which have traditionally been overlooked. Secondly, the study utilizes the nonlinear autoregressive distributed lag technique (NARDL) and two-stage least squares estimate method (2SLS) to provide accurate results despite limited data, addressing both short- and long-run imbalances.

The overarching goal of the study is to address fundamental obstacles to efficient and intelligent energy use, aiding private investors, governments, and financial stakeholders in decision-making and goal-setting. Finally, the research identifies legislative and financial barriers as primary obstacles to achieving energy savings and green energy possibilities, highlighting the need for comprehensive measures to leverage green energy potential.

This paper's remaining sections are structured as follows. Section 2 provides both a practical and theoretical overview of the literature. In Section 3, details of the research techniques are described along with methodology, in Section 4, the findings, analysis, and discussions are given, and in Section 5, the study's conclusion and implications are drawn.

## 2 Literature review

Numerous studies have examined the correlation between rising GDP and rising CO2 pollution [[14], [15], [16], [17]]. Researches have studied the relationship of economy and carbon emissions in South Asian countries [18], lower-middle-income countries [19], upper-middle-income countries [20], and high-income countries [21]. It was concluded that carbon emissions have decreased over time. However, numerous case studies have demonstrated the opposite to be true. For instance Ref. [22], found that between 2001 and 2014, CO2 emissions rose in 46 countries across sub-Saharan Africa. System Generalized Method of Moments (GMM) analysis was used to more accurately assess the correlations in Acheampong's research because it deals with the endogeneity problem [23]. The growth of the finance sector was found to be the third most important factor in carbon pollution after industrialization and economic expansion in a case study conducted in Turkey [24]. Twenty-four MENA countries in a similar situation as those in the study did not correlate financial metrics (the share of domestic loans to the private sector as a percentage of real GDP) and CO2 reduction [25]. In addition, it is necessary to demonstrate the crucial connection between economic growth and carbon pollution. The only time financial factors can help with pollution reduction in a medium-sized economy during financial deregulation and financial sector growth. Strategies to increase financial development in these countries are desperately required because financial variables affect carbon reduction less than other output factors like per head income. Since “financial development” can mean so many different things to many people, the effects of various financial variables on carbon dioxide releases vary widely. The scale and market value of openly listed businesses and the efficacy and amount of stock trading are the most significant factors in reducing CO2 emissions. Still, they are even more essential for averting carbon reduction [26]. Financial scale development raised emissions, but financialization and effectiveness decreased emissions embedded in trade goods and services, according to a second research examining the relationship between financial development and trade-linked carbon emissions [27]. The density of CO2 pollution and energy usage has decreased with financial growth. There has been a reduction in the intensity of carbon emissions even though the carbon intensity has been noted to increase in some local Chinese regions while dropping drastically in others. According to Ref. [28], promoting China's financial growth to cut carbon pollution is a good idea. The current study has used varying meanings of monetary factors and various data collection and methods, which may help explain the variety of results [29]. used bank savings to GDP to measure financial growth, while [30] used the ratio of financial organizations' lending to GDP. Time series data was used by Refs. [31,32] to create a panel dataset through the application of geographic econometrics. Similar to how financial growth through various means affects environmental quality, the issue is whether or not green finance can improve environmental standards. Several studies have made valiant attempts to address this issue. However, the market penetration and environmental impact of green finance need to be introduced. Through the equity market and credit markets, as well as other direct financing mechanisms, environmentally conscious companies can fully utilize social capital. Additionally, green stocks are assisting in increasing green investment, improving companies' capacity to function in a greener manner, and paving the way for the continued growth of the clean technology sector. On the other side, green companies and projects can get bank financing. Second, green finance will influence carbon pollution through the distribution of resources. Green finance aids the green, low-carbon sector on the ground by providing more money and fewer restrictions on borrowing. Eco-friendly financing reduces the price of supporting companies that safeguard the environment, increases the amount of money going to these companies, and promotes the growth of these low-carbon companies. Green high-tech companies carry both risk and profit, making them ideal candidates for ecological funding. Green funding can help integrate and spread hazards associated with more conventional forms of financing. This is so because it can ensure the long-term success of green, low-carbon companies by combining various green finance options [33]. point out that enacting a green credit strategy will restrict the amount of credit available to damaging businesses. As money moves toward low- and no-carbon options, lending standards will constrict for damaging companies. Heavy offenders must find new ways to develop and adapt to attract investors and reduce their environmental impact. Finally, green funding can enhance environmental quality by encouraging innovative practices in the private sector. Under the direction of relevant green finance laws, “green financing” boosts the movement of financial resources to ecologically beneficial, low-carbon companies [34]. The capacity of companies to develop in the green industry is predicted to increase due to green finance laws, according to several studies [35]. Green innovation will loosen budgetary constraints for businesses, letting more money into the industry. This link will improve the green invention skills of businesses, lower pollution levels at companies, and boost business output. However, green finance makes it even more challenging for big toxic businesses to secure financing due to the increased regulatory burden they face. To attract more capital and reduce their detrimental impact on the climate, businesses that produce a lot of waste should prioritize the development of green, low-carbon goods and green, low-carbon technology [36]. Green finance-related steps will improve the creative production and effectiveness of considerably damaging businesses, claim [37]. According to Ref. [38], green finance-related policies will motivate big offenders to implement environmentally friendly advances. This analysis has uncovered three unfilled areas of study. You care about the thing that's being looked into. Few studies have examined the relationship between green funding and carbon emissions. China and India, two of the world's fastest-growing countries, are responsible for a sizable share of the globe's carbon pollution. The “green economic complexity index” (GEPI) is gaining traction in the general consciousness and deserves immediate attention. Therefore, we developed the first-ever measure of green economic success using data from official sources. The third mystery concerns the study methodology. Researchers who have looked at long-term equilibrium connections have been able to identify ties using the CS-ARDL model and the D-H panel causation.

## 3 Methodology and statistics description

### 3.1 Data description and preprocessing

During the period spanning from 1988 to 2022, we collected yearly data employing time series approaches for the E7 countries. The selection of the time frame for data collection was based on the availability and adequacy of data for the specific variables of interest. To accomplish this, we leveraged the World Bank's Development Indicators (WDI) and accessed the global data website to gather comprehensive data on the E7 nations over the specified timeframe.

All forms of renewable energy, encompassing compact, biofuel, hydro, wind, and biogas, were considered in computing the total renewable energy (RE) contribution, expressed in terabyte joules (TJ). The BP Statistics database [39] served as the primary source for the RE data. Additionally, economic and green payment information, crucial for our analysis, was obtained from the International Monetary Fund [40], recognized as the most reliable source in this context.

Gross domestic product (GDP) was utilized as a proxy for income, a common practice in such analyses [41].

To provide a detailed description of the data analysis methodology, we employed rigorous statistical techniques. Firstly, we conducted descriptive statistical analysis to explore the characteristics of the dataset. Secondly, we employed time series analysis techniques to assess trends and patterns over the specified time period. Thirdly, we utilized regression analysis to examine the relationships between variables of interest, such as renewable energy, export diversification, and GDP. Lastly, we employed econometric modeling techniques, including panel data analysis, to account for potential heterogeneity among the E7 countries.

This comprehensive approach ensured robust analysis and reliable findings, allowing us to draw meaningful conclusions regarding the dynamics of renewable energy, export diversification, and economic growth within the E7 nations.

In our analysis, we thoroughly considered various definitions and identified fundamental data gaps. We meticulously evaluated all available data to indicate potential trends and conducted an in-depth examination of significant barriers to implementation.

**Fig. 1:**
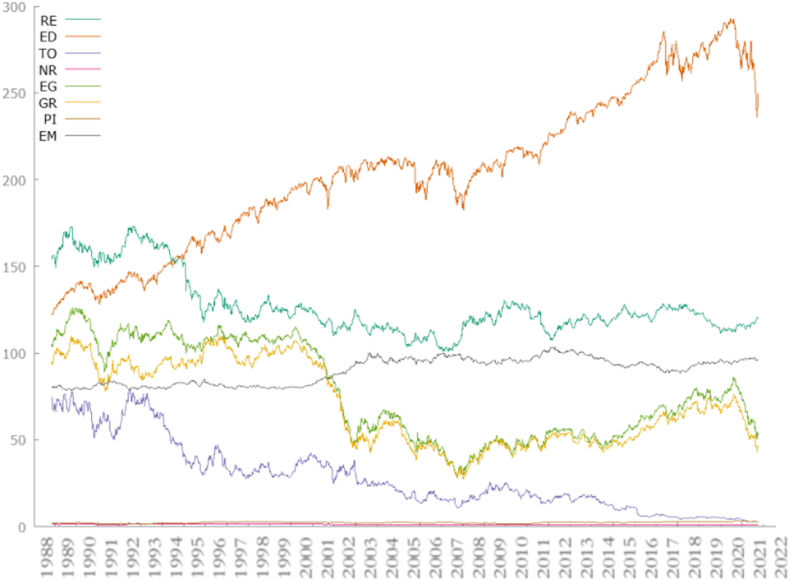
Temporal variation of input data variables showing changes in variables over time (1988–2022).

Fig. 1 illustrates the temporal evolution of input data variables, depicting changes in these variables over the period from 1988 to 2022. This visualization aids in understanding the trends and patterns observed in the data over time, facilitating a comprehensive analysis of the dataset.

Our data analysis involved a multifaceted approach, encompassing descriptive statistics, trend analysis, and identification of key patterns and variations over time. By employing rigorous analytical techniques, we were able to discern meaningful insights and draw robust conclusions regarding the dynamics of the variables under investigation. Table 1 presents the Statistics of data.

**Table 1:** Statistics of data.

Variables	Observation	Avg value	Std.Dev.	**Min**	**Max**	Skew.	Kurt.
Renewable energy (RE)	421	21.88	1.314	23.87	1.314	21.88	2.314
Export diversification (ED)	421	2.41	2.999	4.41	1.102	2.41	1.524
Extensive margin (EM)	421	1.21	1.369	3.21	1.369	1.21	2.369
Green Recovery (GR)	421	2.866	1.698	3.86	1.698	2.86	2.698
Economic growth (EG)	421	29.211	1.321	21.21	1.321	22.21	3.321
Power Industry (PI)	421	5.111	1.987	3.11	1.987	3.11	3.987
Trade openness (TO)	421	2.123	1.654	4.12	1.654	2.12	4.654
Natural resources (NR)	421	−1.654	0.547	−1.65	2.547	−1.654	3.547

Significant advantages of sustainable power include reducing greenhouse gas emissions and increasing energy supply, and renewables are becoming an increasingly important part of the global energy mix. Quantitative evidence was scant, but the bottom quarter of the population seemed to bear the brunt of the natural resources rent (0.01 > p 0.05). The deteriorating impact was most significant in the middle quartile. The lowest quartile, on the other hand, had no longer substantial detrimental effects from TNR. Thus, TNR's negative impacts on IRE were most significant in the center of the distribution. Natural resource rents have a significant impact on investments in renewable electricity. Natural resources like gas, oil, and minerals may impact electricity produced from renewable sources. To reach Asia's decarbonization deployment goals, more has to be done to properly manage the adverse impacts of normal capital, such as government tenancies, tenancies of particular commercial benefits, and a deficiency of financial incentive organizations for variation. High normal investment rental is the most important barrier to investing in renewable energy sources. Previous findings [42] are congruent with our findings.

**Fig. A1:**
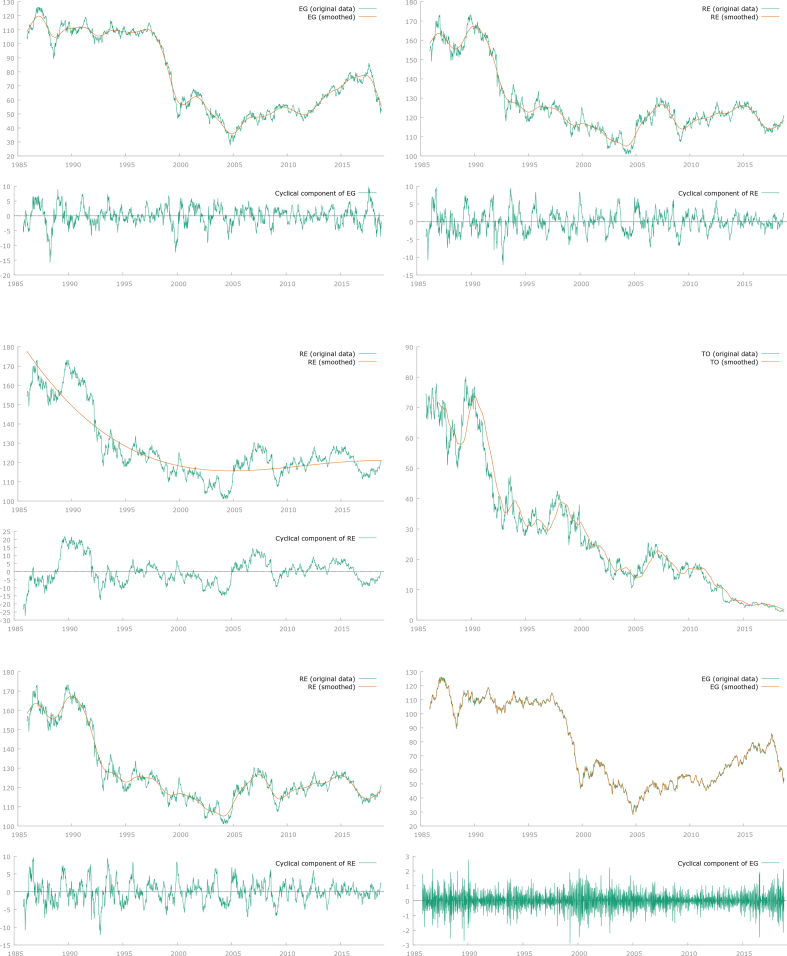
Plot of smooth and normal data.Note: this plot shows how much noise has been removed from each variable during preprocessing stage using MATLAB curve fitting tool box utilizing Savitzky-Golay filter.

**Fig. B1:**
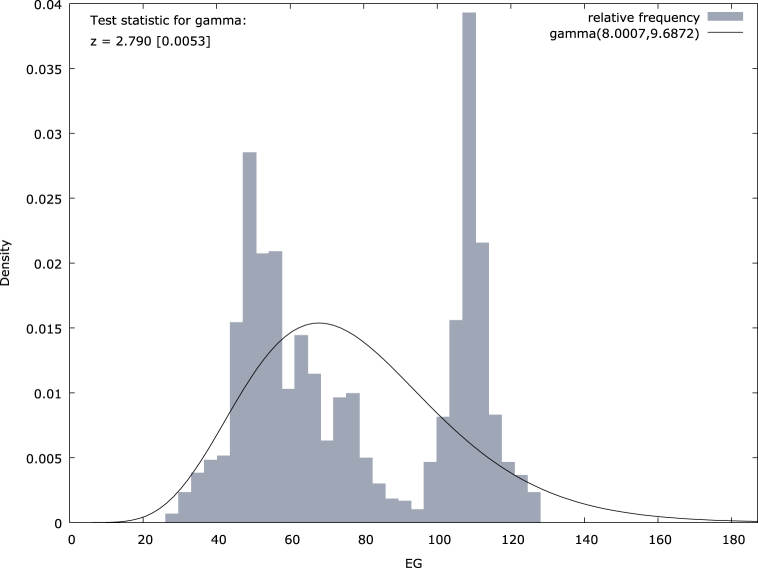
Frequency distribution of Economic growth against gamma distribution.

**Fig. B2:**
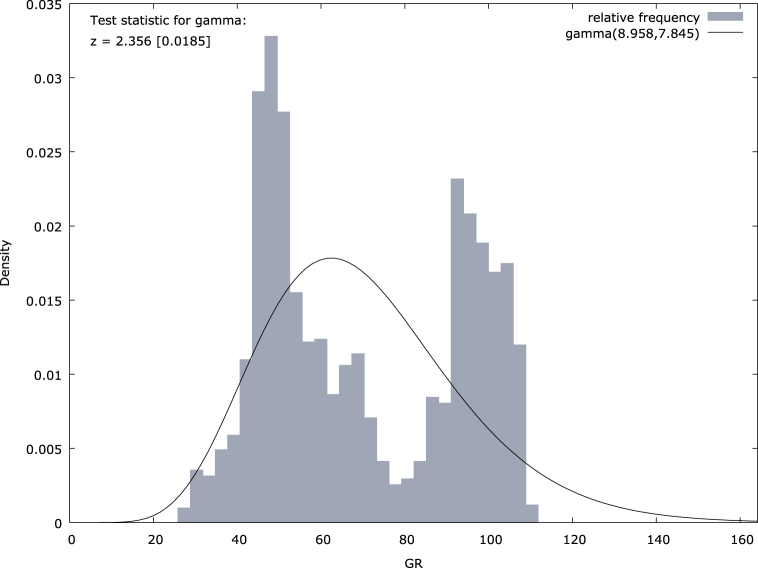
Frequency distribution of green recovery against gamma distribution.

In order to enhance the accuracy of results by reducing noise from the data, pre-processing was conducted using the MATLAB curve fitting toolbox, employing the Savitzky-Golay filter. The figures illustrating the smoothed data and the original data are provided in Appendix-A. Additionally, a comprehensive analysis of the input dataset was carried out to glean valuable insights. However, not all details of this analysis are included in the current study, as the focus is primarily on the implementation aspect. Detailed analyses in the form of plots and tables can be provided upon request. Analysis using the following features/plots for each variable has been undertaken:

• Plot of smooth and normal data shown in Appendix A (Figure A1).
• Frequency distribution plot (frequency distribution against gamma distribution is shown in appendix B) (Figure B1, Figure B2, Table B1 and Table B2)
• Scatter plot with least square fit
• Estimated density
• Frequency spectrum plot
• Time spectrum plot
• Auto-Correlation and Partial Auto- Correlation plots

### 3.2 Methodology

#### 3.2.1 Panel co-integration

Panel co-integration [43], autoregressive distributed lags [44], and unit root tests were employed to assess data stationarity in this econometric investigation. These techniques are instrumental in identifying the underlying causes of unit issues, even in the presence of cross-sectional dependence stemming from provincial globalization.

In the full specification for the E7 nations [45], co-integration was utilized, reflecting one of our top three preferred specifications. This approach aligns with recent studies on renewable energy, employing an identical empirical methodology. These rigorous analytical techniques were pivotal in ensuring robustness and reliability in our data analysis process. The following are the econometric criteria as in Eq. (1), Eq. (2) and Eq. (3).

(1) $$\sum k\Delta {\text{REC}}_{t}\frac{1}{4}a^{3}x\sum i=\text{ki}\frac{1}{4}1\beta_{3i}\Delta \gamma i\rho \sum \text{izi}\frac{1}{4}{REC}_{T}1\varepsilon \mu 3_{i}\Delta Z_{t}4i\rho$$

(2) $$\Delta Z_{t}\frac{1}{4}a^{4}\rho \sum i=\text{ki}\frac{1}{4}1\beta_{3i}\Delta Z_{t}i\rho \sum \text{iki}\frac{1}{4}14_{i}\Delta y_{t}i\rho$$

(3) $$\sum ki{\text{GDP}}_{t}1\mu^{4i}\Delta x{xt}_{t}\sum \text{iki}\frac{1}{4}1\beta_{3i}\Delta \gamma i\rho \sum \text{izi}\frac{1}{4}{TRADEe}_{T}1\varepsilon \mu 3_{i}\Delta Z_{t}\varphi 5i\rho$$

We conducted an examination of the impact of market expansion and goods development on stock market globalization, discerning that the latter exerted a greater influence on export success compared to the former. Our analysis involved modeling the relationships among lubricant value, lubricant, occupation balance, price index inflation (t), and GDP (GDP). Given the significance of the E7 nations within the framework of developing economies, we streamlined the VAR parameters into a simplified form.

For the entire sample of E7 nations, we employed the feasible generalized least squares (FGLS) estimator and fully modified least squares (FMOLS) methods to analyze the magnitude and direction of variables. Each model included control variables and predictors in their specifications, utilizing both FGLS and FMOLS techniques. These methods offer numerous advantages, including the generation of reliable empirical findings, mitigation of reverse causality, and elimination of model endogeneity.

The FMOLS method, widely utilized in the energy-environment literature across both developed and developing countries, including China [46], has been employed to ensure robustness in our analysis. By leveraging these advanced econometric techniques, we aimed to provide comprehensive insights into the dynamics of market expansion, goods development, and stock market globalization within the context of the E7 nations.

## 4 Results and analysis

### 4.1 Primarily findings

Based on our analysis, the findings reveal a positive association between GDP and investment in renewable energy (IRE) across all four quarters. Notably, there exists a substantial correlation between GDP and IRE across the bottom, middle, and top quartiles, albeit it weakens for the higher quartiles. This underscores that higher economic output correlates with increased investment in renewable energy within the E7 countries. The probability of government support for renewable energy sources rises concomitantly with economic growth, indicating that renewable energy is perceived as a high-potential industry requiring significant capital to thrive.

Furthermore, GDP has a significant impact on both the poorer and upper quartiles of the population. Specifically, there is a statistically significant positive influence of GDP on IRE in the bottom quartile, with the effect diminishing in the top 25 %. This suggests that the lowest quartile of GDP exerts the most significant influence on IRE. These findings are consistent with prior research [47], affirming that countries with flourishing economies are more inclined to invest in renewable energy, and the economic outlook of a country plays a pivotal role in the long-term success of renewable energy endeavors.

Table 2 delineates the cost of renewable energy sources, revealing economic growth as a major concern. These results underscore the causal relationship between the economic and financial growth of the E7 nations and investments in renewable energy. Such insights are crucial for policymakers and stakeholders in formulating strategies to foster sustainable economic growth and energy development.

**Table 2:** Outcomes of CIPS and ADF testing.

Tests	Selected Variables	Levels	First Difference
	Renewable energy (RE)	−2.654	−2.123***
	Export diversification (ED)	−3.123	−3.632***
CIPS test	Extensive margin (EM)	−2.789	−4.541*
	Green Recovery (GR)	−2.332	−4.154***
	Economic growth (EG)	−2.369	−3.887***
	Power Industry (PI)	−3.147	−3.111***
	Trade openness (TO)	−3.778	−2.998***
	Natural resources (NR)	−3.887**	−1.332***
	Renewable energy (RE)	5.114**	−21.114***
	Export diversification (ED)	−2.332**	−7.665***
	Extensive margin (EM)	−2.114	−8.112***
Fisher Type ADF	Green Recovery (GR)	−1.997	−1.332***
	Economic growth (EG)	3.112	−9.112***
	Power Industry (PI)	−1.112**	−9.121***
	Trade openness (TO)	−1.121**	−9.133***
	Natural resources (NR)	−2.321**	−9.455***

However, it is imperative to acknowledge certain limitations of our study. Firstly, the analysis relies on aggregated data, which may obscure nuances within individual countries. Secondly, while we have identified correlations between GDP and IRE, causality cannot be definitively established. Future research could delve deeper into these relationships and explore potential moderating factors to provide more nuanced insights.

### 4.2 Stationary tests

In assessing the stationarity of panel data variables, it is essential to ensure accurate regression modeling outcomes, particularly when dealing with nonstationary variables. We employed the five-panel unit root tests [48] to ascertain the stationarity of the variables in our analysis. All statistical analyses were conducted under the assumption of a unit root.

**Fig. 2:**
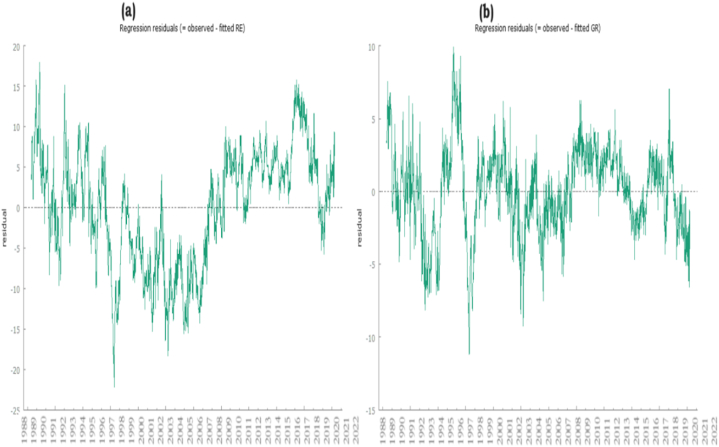
(a & b). Residual plots of Renewable energy and green recovery. (For interpretation of the references to colour in this figure legend, the reader is referred to the Web version of this article.)

Table 3 presents the results of the panel unit root tests for each income bracket, incorporating multiple variables such as renewable energy certificates, gross domestic product, and green recovery. Meanwhile, Table 4 provides the values of the unit root test. Additionally, Fig. 2 (a & b) depicts residual plots of renewable energy and green recovery.

**Table 3:** Panel unit test.

	Panel Modified Phillips-Perron statistics	Breitung t-stat	Panel Phillips-Perron statistics	ADF-Fisher Chi-square	PP- Fisher Chi-square
Renewable energy (RE)	1.123	−1.512	6.412	39.478	69.123***
Export diversification (ED)	−39.12***	−19.11***	−29.11***	1354.19***	1454.09***
Extensive margin (EM)	−1.321**	2.321	1.321	69.36	49.369
Green Recovery (GR)	−19.987***	−9.14***	−19.151***	229.11***	665.32***
Economic growth (EG)	3.121	1.133	29.321	21.114	9.33
Power Industry (PI)	−9.112***	−5.444***	−9.321***	255.144***	174.122***
Trade openness (TO)	−1.112	2.147	2.288	66.147	59.147
Natural resources (NR)	−19.321***	−2.178***	−19.147***	144.844***	614.221***
Panel Modified	−39.147***	−19.369	−19.478***	147.144***	8.221***

**Table 4:** The outcomes of unit root test.

		IPS	Fisher-ADF	Fisher-PP
Level	lnFDE	3.4121	39.121	59.141
	lnREC	−3.147***	90.123***	331.114***
	lnGHG	1.1212	49.1212	69.123
	lnNPS	4.1478	19.158	29.147
	lnEP	−3.1478***	114.745***	115.147***
	lnRD	5.147	19.369	29.126
	RDI	1.111	59.123	59.123
	lnGDP	3.332	19.222	19.407
	lnPEU	4.321	19.147	19.654
First difference	lnREC	−3.147***	91.123***	331.114***
	lnWPC	−4.147***	93.123***	331.114***
	LnSO 2	−5.147***	99.123***	321.114***
	lnFDE	−6.1478***	116.745***	125.147***
	lnPEC	−2.1478***	118.745***	135.147***
	lnREC	−6.1478***	119.745***	155.147***
	RDI	−9.1478***	111.745***	165.147***
	lnGDP	−7.1478***	113.745***	175.147***
	lnPEU	−8.1478***	114.745***	195.147***

Our findings reveal that all variables were integrated of order I and were non-stationary at the level but exhibited stationarity at the first difference. This aligns with existing literature on panel data analysis, which emphasizes the importance of considering variable stationarity for robust regression modeling outcomes.

Practically, these results underscore the significance of addressing non-stationarity in panel data analysis to ensure the reliability of empirical findings and inform effective policy-making and decision-making processes.

However, it is crucial to acknowledge certain limitations of our study. Firstly, while we have identified the stationarity of variables, our analysis may be subject to the limitations inherent in panel data analysis, such as potential endogeneity and omitted variable bias. Additionally, the findings may be influenced by the specific econometric techniques employed. Future research could explore alternative methodologies and consider additional factors to provide more comprehensive insights into the dynamics of panel data variables.

### 4.3 Panel regression and GMM methods

We employed the Fully Modified OLS (FMOLS) and System GMM techniques to explore the varying effects of our key variables, following the establishment of a co-integration relationship among them, particularly export diversification and renewable energy. The empirical results of the entire panel specification with the three baseline models are presented in Table 5a, Table 5ba and 5b.

**Table 5a:** Outcomes of econometric approximations.

Variables	Model I		Model II		Model III	
	Eq (I)	Eq (II)	Eq (III)	Eq (3)	Eq (I)	Eq (II)
Renewable energy (RE)	1.478***	1.122***	1.145***	1.987***	1.147***	1.258***
	(1.147)	(1.141)	(1.144)	(1.148)	(1.149)	(1.150)
Export diversification (ED)	1.471***	1.122***	2.145***	3.987***	2.147***	2.258***
	(1.999)	(1.991)	(1.992)	(1.993)	(1.994)	(1.995)
Trade openness (TO)	2.474***	2.120***	1.146***	2.988***	3.141***	1.259***
	(1.112)	(1.321)	(1.654)	(1.651)	(1.325)	(1.325)
Natural resources (NR)	2.484***	2.128***	1.143***	2.987***	3.148***	1.257***
	(1.133)	(1.112)	(1.320)	(1.650)	(1.659)	(1.325)
Economic growth (EG)	−1.11**	1.12***				
	(1.12)	(1.25)				
Green Recovery (GR)		−1.1***				
		(1.14)				
Power Industry (PI)			1.141***	9.121***		
			(1.221)	(1.321)		
Extensive margin (EM)				−19.11***		
Autocorrelation	No	No	No	No	No	No
Homoscedastic	Yes	Yes	Yes	Yes	Yes	Yes

**Table 5b:** Outcomes of model 1 to 3 estimates.

Variables	Model I		Model II		Model III	
	Eq (I)	Eq (II)	Eq (III)	Eq (III)	Eq (IV)	Eq(V)
Renewable energy (RE)	2.484***	2.128***	1.143***	2.987***	3.149***	1.251***
	(1.133)	(1.112)	(1.321)	(1.651)	(1.650)	(1.324)
Export diversification (ED)	2.414**	2.124**	1.147**	2.967**	3.143**	1.259**
	(1.111)	(1.32)	(1.651)	(1.632)	(1.998)	(1.541)
Trade openness (TO)	−2.145**	−1.987**	−1.654	−1.258	−1.741**	−1.321**
	(1.365)	(1.121)	(1.326)	(1.365)	(1.121)	(1.326)
Natural resources (NR)	2.414**	2.124**	1.147**	2.967**	3.143**	1.259**
	(1.365)	(1.191)	(1.327)	(1.361)	(1.129)	(1.328)
Economic growth (EG)	−1.112*	2.480**				
	(1.654)	(2.369)				
Green Recovery (GR)		−1.32***				
		(1.632)				
Power Industry (PI)			2.12	9.32***		
			(1.32)	(5.12)		
Extensive margin (EM)				−29.32**		
						(1.33)
Constant	−1.222**	−2.888**	−7.332**	−9.121*	−2.698**	−2.147*
	(5.321)	(4.654)	(4.987)	(5.321)	(4.654)	(4.987)
Observations	421	421	421	421	421	421
**R**-squared	0.212	0.321	0.145	0.361	0.691	0.321

**Figure (3a & 3b):**
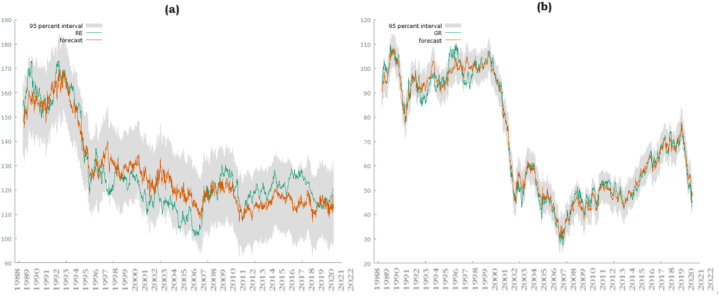
Forecast plots of Renewable energy and green recovery indicating closer integration and association of green recovery with other variables used in this study.

Fig. 3a and Fig. b illustrates the forecast performance of renewable energy and green recovery using other variables in the study. The plots depict the integration and closer correlation of green recovery compared to renewable energy with other variables in the study.

Our findings suggest that economic growth, production modernization, and the availability of natural resources all exert positive and substantial effects on renewable power. Conversely, economic diversification, margin expansion, and margin contraction adversely affect renewable energy utilization. These results are consistent with existing literature on the subject, which underscores the importance of economic factors, production processes, and resource availability in influencing renewable energy outcomes. Practically, these findings have significant implications for policymakers and stakeholders involved in energy planning and sustainable development initiatives.

However, it is essential to acknowledge certain limitations of our study. Firstly, the analysis is based on aggregated data, which may overlook nuances within individual countries or regions. Additionally, while we have identified associations between variables, establishing causality remains challenging. Future research could address these limitations by incorporating more granular data and employing advanced econometric techniques to further elucidate the dynamics of renewable energy utilization.

Increases in efficiency also help when it comes to producing renewable energy. Scientific data shows that ENEF harms renewable energy investment across both deciles. Significant positive correlations among ENEF and IRE were seen across all four quartiles; however, their strengths varied. The favorable effect of ENEF was confirmed by the data in all four deciles (0.1 > p), although it was most pronounced in the mean. The positive benefits of ENEF on IRE were most significant for those in the middle fifty percent.

Renewables provide more significant contributions to renewable energy technology and lower energy consumption. These efficiencies vary considerably depending on market size, energy architecture, geographical availability, and environmental temperature. New information corroborated previous findings. Saving money, reducing carbon emissions, creating employment, and meeting rising power needs can be accomplished quickly and cheaply using efficient electricity. Hence, fuel economy may aid in diversifying a company's resource portfolio and providing insulation from the volatility of fuel costs. As a result, fuel economy has a net-positive impact on the growth of renewable energy sources. The linear regression results show that the statistical evidence decreased when financial performance grew in the top quartile. The mean percentile provides the most robust statistical evidence of the TNR's harmful effect. So, we may infer that the adverse impact of TNR on IRE is significant if the economic future showed no indication of significance. Table 4 shows that economic success's positive effects on IRE and TNR's adverse effects on IRE diminished when a certain threshold was attained. From the bottom to the top quartiles, there was a statistically significant difference in TI and ENEF. After ensuring enough variation in the gradients and dependencies between the panels representing the E7 countries, we checked for normality. Table 4 shows predicted outcomes from the upgraded IPS (CIPS) test developed by Ref. [49]. Tests for data stationarity were performed using the I (0) and I (0) statistics. Leveled information estimates showed that GF, IER, and NRV were significantly significant at 10 % and 5 %, respectively. In contrast, I placed little stock in the EP or TI (0). Normality tests on these variables were performed using I. Thus, EP and TI were both shown to be critically essential at the 10 %, 5 %, and 1 % stages of significance. All variables under consideration were constant at either (I (0) or (I (1), making it possible to test for a cointegrating relationship between them. The economy benefits from investments in renewable energy sources. A rise of 0.352 % (AMG) and 0.1167 % (CCEMG) in financial forecasts and an increase of 1 % in energy investment sources are provided. These are noteworthy at different stages of significance. Consistent with the findings of earlier research [50], it has been demonstrated that increasing energy expenditure improves economic efficiency. The expansion of fossil fuels and renewable energy sources, such as breeze and astral, contribute to the E7 areas' overall economic growth. Although lubricant and gas are substantial sources of CO2 releases in the E7, their use is essential to economic progress. So, Table 5a, Table 5ba and 5b might benefit from a strategy encouraging increased energy source investment. Globalization and trade liberalization shorten the time for goods to move freely across countries. Yet interprovincial commerce existed even before the advent of globalization and international trade. Due to international trade, increased dependence on other nations has led to more significant commodities and good exchange among China's provinces. This dependency's upsides include monetary stability and fast economic growth. The CD test agreed with the results from Ref. [51], demonstrating that all of the investigated variables were statistically significant at different levels. Consequently, we found that the EP, GF, IER, NRV, and TI of the E7 financial prudence were bridge-dependent, thereby rejecting the null hypothesis of no pass reliance. In addition, we estimated co-integration using [52]. We tested for long-run co-integration, with results for the whole sample showing evidence for a stable connection with constant and trend as shown in Table 6.

**Table 6:** Outcomes of Co-integration test for E−7 economies.

**E**-7 Economies	No Deterministic- Specification	With constant	With trend
Explained Variable: Green innovation			
Whole Sample	−3.14***	−4.114***	−2.321***
China	−3.981***	−2.121***	−4.121***
Turkey	−1.299***	−3.654***	−3.012***
Indonesia	−4.121***	−2.654***	−5.125***
Brazil	−3.147***	−3.147***	−3.145***
India	−4.121***	−3.147***	−4.654***
Mexico	−5.121***	−4.654***	−5.321***
Russian	−4.22***	−5.321***	−5.321***

**Notes**: ∗∗∗, ∗∗, ∗ represent level of implication at 1 %, 5 % and 10 %.

### 4.4 Results of CSD and heterogeneity

Due to concerns related to cross-sectional dependence (CSD) and heterogeneity in panel data, our empirical analysis was constrained to second-generation unit root tests such as the CIPS and MCIPS. In Table 7, all variables are found to be stationary at (I (0), indicating that the model's deviation and mean remain consistent over time. These findings suggest that the CIPS test is superior to the approach as it accounts for a broader range of structural breaks and addresses cross-sectional dependence and inconsistency.

**Table 7:** Outcomes of unit root tests with and without physical disruptions.

Variables	Level I (0)		1st Difference I (1)	
	CIPS	M-CIPS	CIPS	M-CIPS
Renewable energy (RE)	−4.654***	−3.122**	–	–
Export diversification (ED)	−2.122***	−2.365**	–	–
Extensive margin (EM)	−2.121***	−6.121**	–	–
Green Recovery (GR)	−4.147***	−2.321**	–	–
Economic growth (EG)	−2.987***	−4.358**	–	–

BaiandCarrion−i−Silvestre						
	Z	Pm	P	Z	P_m_	P
Renewableenergy(RE)	1.987	1.987	19.365	−3.654***	6.698***	49.987***
Exportdiversification(ED)	1.984	1.987	18.365	−4.654***	6.698***	55.987***
Extensivemargin(EM)	2.987	1.987	17.365	−3.654***	4.698***	49.987***
GreenRecovery(GR)	3.989	1.987	16.365	−5.654***	5.698***	51.987***
Economicgrowth(EG)	2.983	1.980	18.365	−6.654***	8.698***	69.987***

Furthermore, when incorporating multiple structural breaks into each variable, the results for several variables demonstrate significant changes. Table 7 presents the average p-values from the modified Sargan Bhargava (MSB) statistical test, where Z represents the adjusted or regular entity statistics. The data indicate that all variables exhibit skewness at a certain level but converge at the first difference.

These findings contribute to the existing literature by highlighting the importance of employing robust unit root tests, such as the CIPS and MCIPS, in panel data analysis to account for cross-sectional dependence and structural breaks accurately. From a practical standpoint, these results underscore the necessity of adopting rigorous statistical techniques to ensure the reliability and validity of empirical findings in econometric research.

However, it is essential to acknowledge the limitations of our study, such as the potential for omitted variable bias and the assumption of exogeneity in panel data analysis. Future research could address these limitations by employing more sophisticated econometric techniques and exploring additional variables to provide a comprehensive understanding of the factors influencing economic phenomena.

It was hypothesized that technological advancement would significantly benefit the economies of the E7 nations under consideration. The AMG and CCEMG saw a 1.117 % and 1.321 % increase in output for every 1 % increase in technical innovation. These results are significant at the 1 %, 5 %, and 10 % levels of statistical analysis. According to Ref. [53], the current research results are consistent with those of earlier studies, suggesting that technological innovation positively affects economic growth. Acceptance of technology and innovation not only increases manufacturing efficiency but also increases tax revenue. Economic development in the E7 area is a result of innovative policies and technical progress, according to Ref. [54], which corroborated the findings of this research. So, as demonstrated in Table 7, technical progress may be a valuable instrument for boosting economic performance and guaranteeing fast economic development. The development of technology has not only helped the economy but has also contributed significantly to environmental restoration. As technology advances, more efficient tools become available to maximize production while minimizing the waste of energy and materials.

### 4.5 Regression analysis

The impact of foreign direct investment (FDI) on nonrenewable and renewable energy sources over the two economic periods is shown in Table 8. The results reveal that foreign direct investment (FDI) has a small but unfavorable direct effect on renewable energy growth during economic downturns. Foreign direct investment did not increase the use of renewable energy sources during the recession. Similarly, during economic downturns, the collaborative effect of FDI showed inefficiency in raising both renewables and conventional energy usage. However, FDI was found to boost green energy amid economic booms. Moreover, FDI promotes the use of fossil fuels. The prosperity that attracts foreign direct investment also increases the need for energy. This need cannot be satisfied solely by renewable energy sources.

**Table 8:** Outcomes of longer run properties.

Long run	lnREC	lnFDE
	FMOLS	DOLS
lnPEC	1.354***	1.698***
	(1.147)	(1.987)
LnGHG	1.114***	1.981***
	(1.147)	(1.321)
lnGDP	2.998***	1.987*
	(1.1478)	(1.985)

Shortrun	LnREC
ecm	−1.984***(1.147)
d.lnPEC	1.965(1.142)
d.lnRD	1.632***(1.654)
d.lnGDP	2.654***(1.321)
Intercept	−1.321***(1.321)

This analysis demonstrates that higher rates of investment in renewable energy (IRE) in the E7 nations are associated with enhanced levels of technical modernization. Upon closer examination of the data, it becomes evident that technological innovation (TI) significantly affects both the lowest and highest income deciles. TI exhibits a statistically significant positive effect in the lower quartiles (p < 0.01). However, as one moves up the income deciles, the favorable impact of TI on IRE gradually diminishes, reaching a minimum in the top quartile. Therefore, individuals in the lower income quartiles (Q 0.25) stand to benefit more from technological innovation in renewable energy. This finding aligns with the suggestion by Ref. [9] that a broader range of technological advancements would encourage more funding for renewable energy.

While several renewable technologies have been developed, their widespread adoption is hindered by reliability and cost concerns. Table 8 compares the impacts of renewable and nonrenewable sources during two economic periods. The results indicate that institutions play a crucial role in influencing people's use of renewable energy sources positively and conventional energy sources negatively. Strong institutions, through the implementation of stringent laws and regulations, can mitigate the adverse effects observed in regression findings and promote growth in the appropriate sectors of the economy. However, it is essential to note that as output grows, there is an increased demand for energy, necessitating the use of nonrenewable resources.

Overall, while institutions have a positive impact on the renewable energy industry's development, their effect on corporate sequences remains negative but marginal. This suggests that while institutions are vital for fostering renewable energy growth, there may be limitations in their ability to influence corporate decisions in this sector.

## 5 Conclusion and policy implications

In view of the impending implementation of green finance and renewable energy sources, this paper investigates the methods by which the E7 countries are working to improve their energy efficiency. Moreover, green financing is a sustainable option for energy conservation projects because it focuses on the economy and the environment. The potential for profit as well as the barriers to entry in the renewable energy and energy efficiency industries were investigated in this study. Specifically, it examined the asymmetrical and nonlinear impacts of greener financing and renewable energy on energy efficacy. The following results The E7 areas are rated according to their differences in the growth of green financing. From highest to lowest: China (1.52 points), Brazil (1.44), India (1.35), Indonesia (1.94), Mexico (1.73), and Russia (1.73 points) (1.93). The Gini coefficient in 2019 was highest in Turkey (1.75) and second highest in Russia (1.35). The study's findings suggest that using renewable energy bases and power efficacy in the E7 countries has a talented but uncertain future. Empirical studies show that the lack for substantial commercial and administrative resources in the energy business is a significant barrier to expanding access to energy, increasing energy safety, and encouraging environmentally maintainable financial growth. The financial potential of energy efficiency is examined in this research with an eye on assisting E7 countries in overcoming barriers to greener financing and renewable power strategies. Increasing public financing and luring private-sector investment in climate-resilient infrastructure requires a more favorable legislative and regulatory environment. A more coordinated and combined provincial, cross-border, and multi-country cooperation on climate change-related problems across Asian countries is urgently needed to lower policy obstacles. For excellent population coverage, improved climate effect, and more poverty reduction, it is crucial that the government develop adequate protocols for channeling climate financing to local actors. As a result of these results, it is recommended that environmental legislation and expenditures be made by introducing new, green technologies and renewable energy sources. Finding ways to enhance the utilization of renewable energy sources is crucial. We need to think about how to export diversification will affect the utilization of renewable energy since international commerce is an inevitable aspect of globalization. Export diversification has the potential to serve as a strategy tool for reducing the use of nonrenewable energy sources. In particular, distribute variation might become a crucial strategy to enhance renewable power, adding to these results from our research. Findings from this study should inform the development of environmental policies and investments to promote technological innovation using renewable energy sources.

### 5.1 Policy implications

This investigation's results lead to several new, applicable conclusions. Regarding reducing carbon emissions and improving sustainability, the government should prioritize adopting inventions that achieve both. Increasing spending on renewable infrastructure, expanding export options, and creating innovative new products are all viable strategies for achieving this goal. Second, administrations in industrialized and developing nations must provide financial incentives for renewable energy development through investment subsidies, tax exemptions, and rebates. There are caveats to this research that must be recognized. The research began by looking at five critical markers of climate change, which opened the door for the individual selection of 14 nations. The statistical impact of each element on climate investment potential and money flow has to be investigated in future research. Second, it's not certain that easing barriers to private investment in renewable energy and energy efficiency projects and programs would improve people's access to these resources and sustain their livelihoods. Whereas private companies aim to maximize profits, public agencies prioritize society's and the environment's well-being. There are gaps in this study's methodology that future investigators might explore. For starters, because the sample size of this research is so tiny (just E7 nations), there may be discrepancies in the findings when applied to other economies. Public-private partnerships and customer-friendly policies for industries and enterprises may increase the market share of renewable energy and encourage its usage by citizens. Lastly, the commercial sector's uptake of renewable energy technology via purchases is a crucial channel for expanding that sector's share of total energy consumption. Limits imposed by policymakers might force businesses to operate on a larger scale.

## Ethics approval and consent to participate

Not applicable.

## Consent for publication

All of the authors consented to publish this manuscript.

## Funding

Funding information is not available.

## Data availability

We collected relevant data from World Bank open data available at https://data.worldbank.org/. For any further query on data, corresponding author at email address *13995316589@163.com* may be approached.

## CRediT authorship contribution statement

**Mingda Shi:** Writing – review & editing, Writing – original draft, Visualization. **Jing Yu:** Writing – review & editing, Writing – original draft, Investigation, Data curation, Conceptualization.

## Declaration of competing interest

The authors declare that they have no known competing financial interests or personal relationships that could have appeared to influence the work reported in this paper.

## Figures and Tables

**Table B1:** Frequency distribution of Economic growth against gamma distribution

interval	midpt	frequency	rel.	cum.
<29.636	27.880	4	0.23 %	0.23 %
29.636–33.148	31.392	14	0.81 %	1.05 %
33.148–36.660	34.904	23	1.34 %	2.39 %
36.660–40.172	38.416	29	1.69 %	4.07 %
40.172–43.685	41.929	31	1.80 %	5.88 %
43.685–47.197	45.441	93	5.41 %	11.29 %*
47.197–50.709	48.953	172	10.01 %	21.30 %***
50.709–54.221	52.465	125	7.28 %	28.58 % **
54.221–57.733	55.977	126	7.33 %	35.91 % **
57.733–61.245	59.489	62	3.61 %	39.52 % *
61.245–64.757	63.001	87	5.06 %	44.59 % *
64.757–68.270	66.514	69	4.02 %	48.60 % *
68.270–71.782	70.026	38	2.21 %	50.81 %
71.782–75.294	73.538	58	3.38 %	54.19 % *
75.294–78.806	77.050	60	3.49 %	57.68 % *
78.806–82.318	80.562	30	1.75 %	59.43 %
82.318–85.830	84.074	18	1.05 %	60.48 %
85.830–89.343	87.586	11	0.64 %	61.12 %
89.343–92.855	91.099	10	0.58 %	61.70 %
92.855–96.367	94.611	6	0.35 %	62.05 %
96.367–99.879	98.123	28	1.63 %	63.68 %
99.879–103.39	101.64	49	2.85 %	66.53 % *
103.39–106.90	105.15	94	5.47 %	72.00 % *
106.90–110.42	108.66	237	13.80 %	85.80 % ****
110.42–113.93	112.17	130	7.57 %	93.36 % **
113.93–117.44	115.68	50	2.91 %	96.27 % *
117.44–120.95	119.20	28	1.63 %	97.90 %
120.95–124.46	122.71	22	1.28 %	99.19 %
≥ 124.46	126.22	14	0.81 %	100.00 %

Test for null hypothesis of gamma distribution:z = 2.790 with p-value 0.00528.

Frequency distribution for EG, obs 1-1718.

number of bins = 29, mean = 77.5045, sd = 27.4007.

**Table B2:** Frequency distribution of green recovery against gamma distribution

interval	midpt	frequency	rel.	cum.
<28.822	27.340	5	0.29 %	0.29 %
28.822–31.786	30.304	18	1.05 %	1.34 %
31.786–34.751	33.269	16	0.93 %	2.27 %
34.751–37.715	36.233	25	1.46 %	3.73 %
37.715–40.679	39.197	30	1.75 %	5.47 %
40.679–43.644	42.161	56	3.26 %	8.73 % *
43.644–46.608	45.126	148	8.61 %	17.35 % ***
46.608–49.572	48.090	167	9.72 %	27.07 % ***
49.572–52.536	51.054	141	8.21 %	35.27 % **
52.536–55.501	54.019	79	4.60 %	39.87 % *
55.501–58.465	56.983	62	3.61 %	43.48 % *
58.465–61.429	59.947	63	3.67 %	47.15 % *
61.429–64.394	62.911	44	2.56 %	49.71 %
64.394–67.358	65.876	54	3.14 %	52.85 % *
67.358–70.322	68.840	58	3.38 %	56.23 % *
70.322–73.286	71.804	36	2.10 %	58.32 %
73.286–76.251	74.769	21	1.22 %	59.55 %
76.251–79.215	77.733	13	0.76 %	60.30 %
79.215–82.179	80.697	15	0.87 %	61.18 %
82.179–85.144	83.661	21	1.22 %	62.40 %
85.144–88.108	86.626	43	2.50 %	64.90 %
88.108–91.072	89.590	41	2.39 %	67.29 %
91.072–94.036	92.554	118	6.87 %	74.16 % **
94.036–97.001	95.519	106	6.17 %	80.33 % **
97.001–99.965	98.483	96	5.59 %	85.91 % **
99.965–102.93	101.45	86	5.01 %	90.92 % *
102.93–105.89	104.41	89	5.18 %	96.10 % *
105.89–108.86	107.38	61	3.55 %	99.65 % *
≥ 108.86	110.34	6	0.35 %	100.00 %

Test for null hypothesis of gamma distribution:z = 2.356 with p-value 0.01846.

Frequency distribution for GR, obs 1-1718.

number of bins = 29, mean = 70.2754, sd = 23.48.

## Appendix A

Figures below indicate the smoothing of data vs actual data.
